# Temporal variation in black-caiman-nest predation in varzea of central Brazilian amazonia

**DOI:** 10.1371/journal.pone.0183476

**Published:** 2017-08-30

**Authors:** Kelly Torralvo, Robinson Botero-Arias, William E. Magnusson

**Affiliations:** 1 Programa de Pós-Graduação em Ecologia, National Institute of Amazonian Research; Manaus, AM, Brazil; 2 Mamirauá Institute for Sustainable Development, Tefé, AM, Brazil; 3 Department of Wildlife Ecology and Conservation, University of Florida, Gainesville, FL, United States of America; 4 Coordenação da Biodiversidade, Instituto Nacional de Pesquisas da Amazônia; Manaus, AM, Brazil; James Cook University, AUSTRALIA

## Abstract

On the Amazon floodplain, the main predators of black caiman (*Melanosuchus niger*) eggs are jaguars (*Panthera onca*), tegu lizards (*Tupinambis teguixim*), capuchin monkeys (*Sapajus macrocephalus*) and humans (*Homo sapiens*). In this study, we investigated the relationship between predator attacks on nests and incubation period, and evaluated the influence of initial predation on subsequent predation in the Mamirauá Sustainable Development Reserve. We also evaluated the influence of presence of females near the nests and manipulation of nests on the occurrence of attacks. We compared results from data obtained with camera traps and vestiges left by predators on estimates of rates of predation by different predators. Egg predation was recorded in 32% of the 658 black caiman nests monitored during two years. Our results suggest that the probability of predation on black caiman eggs is relatively constant throughout the incubation period and that predation on eggs was lower when adults, presumably females, were present. Careful opening of nests and handling of eggs did not increase the number of attacks on black caiman nests. Nest opening by a predator appeared to increase the chances of a subsequent attack because most of the attacks on nests occurred soon after a predator first opened the nest. However, attacks by another species of predator do not appear to be necessary to initiate attacks by any other species of predator. Results based on camera traps and vestiges differed, but use of vestiges was adequate for identifying the principal predators on eggs in black caiman nests and, in many circumstances, the vestiges may be better for estimating predation by humans. In this study, opening nests and handling eggs did not increase the number of attacks on black caiman nests.

## Introduction

Susceptibility of reptile and bird nests to attacks by predators may vary with incubation phase and parental behavior [1,2]. On the Amazon floodplain, the main predators of black caiman (*Melanosuchus niger*) eggs are jaguars (*Panthera onca*), tegu lizards (*Tupinambis teguixim*), capuchin monkeys (*Sapajus macrocephalus*) and humans (*Homo sapiens*) [3,4]. However, it is not known if the intensity of attacks by predators varies throughout the incubation period or whether some nests are more vulnerable than others.

Black caimans nest annually in the dry season (from September to January in Central Brazilian Amazonia) and the incubation period can extend up to 90 days [5,6]. The second most frequent cause of egg mortality after predation is nest flooding [3,7], which occurs at the end of the incubation period. Nests of black caiman are mostly located in flooded forests (varzea) around isolated water bodies where the water level rises later in the season [8].

The black caiman is widely distributed in the Amazon basin, but occurs most frequently in varzea in sympatry with spectacled caimans (*Caiman crocodilus*). Female spectacled caimans nest in the same period and same general area as black caimans [8]. The main predators of spectacled caiman eggs are also tegu lizards, capuchin monkeys, jaguars and humans [9]. Spectacled caimans may nest up to hundreds of meters from water bodies and often attend the nest over the whole incubation period, far from water and without feeding [4,8,10,11]. Unlike the spectacled caiman, black caiman females usually nest near water bodies and remain in the water most of the time [4,7,12].

Black caimans produce up to 60 eggs per clutch [6,7] and several events of predation involving different species of predators can occur in a single nest. In other species, the behavioral response of the prey to reduce the action of a predator may facilitate the action of a second species [13,14]. In the case of nest predation, the action of the first predator can act as a facilitator to the foraging of a second predator by exposing the eggs.

Black caiman nests are mounds of earth, leaves and sticks. Predators attacking nests leave characteristic vestiges, such as holes, scattered shells and footprints. These have been used to identify egg predators of black and spectacled caimans [4,9]. However, it is unknown if these records allow the correct identification of predators. More precise data have been obtained by the use of camera traps for nests of other species of crocodilians [15,16].

Predator attacks on caiman nests can also be influenced by research activities carried out during the incubation period. Studies have shown an increase of up to 70% in attacks on nests of other caiman species that were exposed to human disturbance, such as opening nests or capture of females [9,17,18].

In the present study, we investigated the following questions: (1) Does the probability of egg predation on black caiman nests vary throughout the incubation period? (2) Does the proportion of time that females attend nests affect the probability of predation? (3) Does predation by one species of predator influence predation by other species? (4) Do the proportions of nests attacked by different predators estimated from records of vestiges reflect the proportions of nests effectively attacked by those predators? (5) Does opening nests and handling of eggs for research purposes make them more vulnerable to predation?

## Material and methods

**Fig 1:**
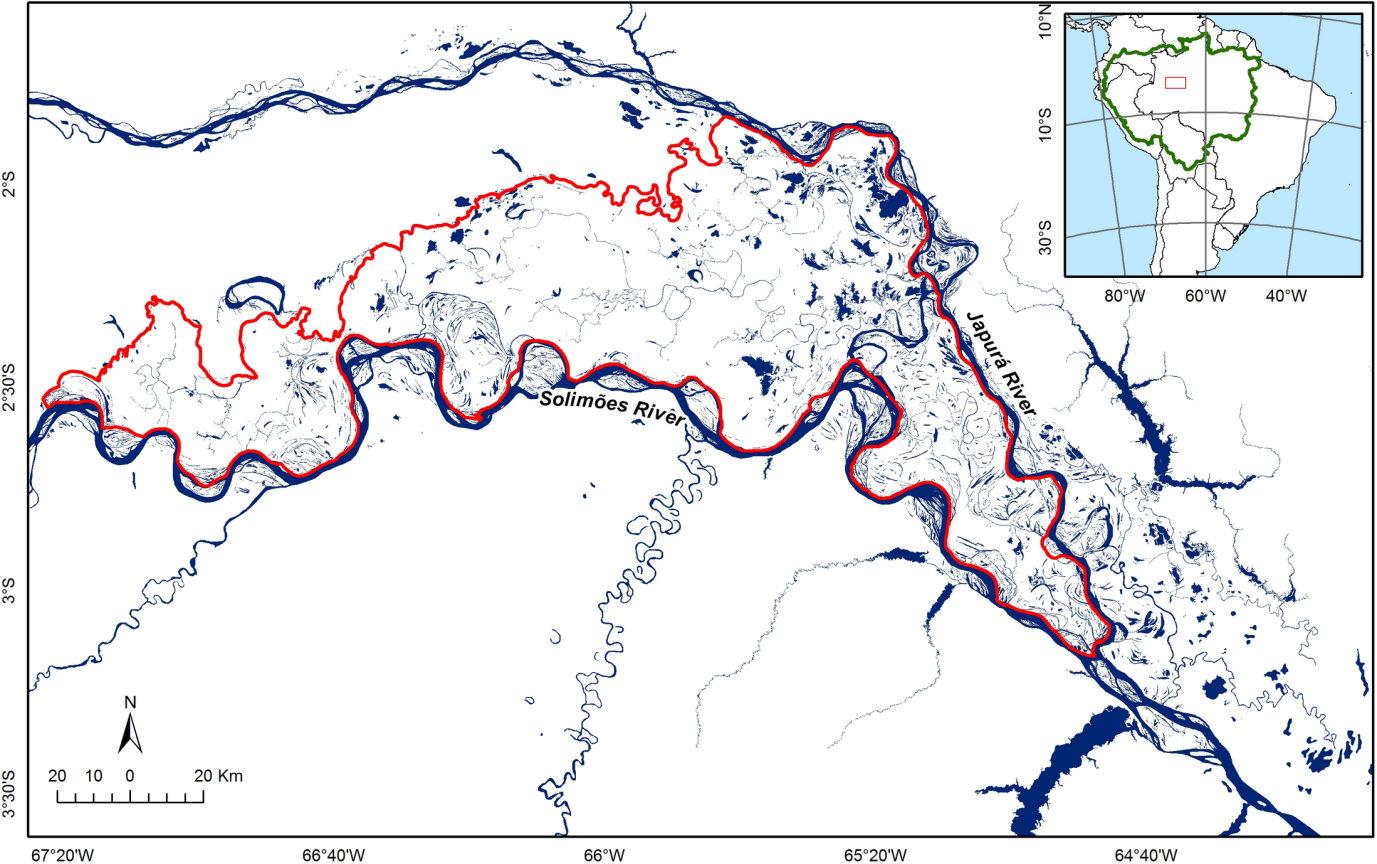
Location of the study area. Red lines show the limits of the Mamiraua Sustainable Development Reserve–MSDR. The green line on the inset indicates the limits of the Amazon basin. Map created by Jefferson Ferreira Ferreira.

The study was conducted in October, November and December of 2013 and 2014 in the Mamirauá Sustainable Development Reserve (MSDR) located in Central Brazilian Amazonia between the Amazon (Solimões) and Japurá rivers (Fig 1). The reserve is covered by varzea habitats and subject to a large monomodal flood pulse of up to 10 m in amplitude [19].

Nest searches were undertaken on foot or from small boats in the vicinity of 288 water bodies, mainly lakes, and the locations of nests were recorded with a GPS model Garmin 76CSx®. Identification of predators was based on vestiges for 595 nests and on records from camera traps in 63 nests. Vestiges were not recorded for the nests with camera traps.

Evidence of predation, such as holes in the nest, missing eggs, scattered shells and footprints near the nest, were used to identify predators that attacked nests monitored without camera traps. Camera-traps (model PC800 Reconyx®) were attached to trees about 0.40 cm above ground and at a mean distance of 1.41 m (0.70–2.80 m) from nests, so that the entire nest was captured in the images. The cameras were programmed to take five pictures at 10-s intervals, for as long as the camera sensor identified movement, and photos were downloaded every 15 days. In most cases, the nests were monitored with camera traps shortly after they were built (estimated at ≤10 days n = 19, ≤20 days n = 21 and >20 days n = 23, from the date used here as the earliest probable nest construction) until the end of the nesting period. If all eggs in a nest had been removed by predators, the camera trap was installed on another nest without evidence of predation in the same lake. Nests were visited from one to six times, and the presence or absence of a caiman, presumably the female, near the nest was recorded on all visits.

Fourteen of the nests monitored by camera traps were opened for counting and measuring eggs. This procedure was part of other research activities and involved manual opening of the nest, removal and handling for measurements of eggs, replacement of eggs, and nest closure.

Entry permission to the Mamiraua Sustainable Development Reserve was granted by the Instituto de Desenvolvimento Sustentável Mamirauá. This is study is included in the authorization for scientific activities number 46635–2 of the Biodiversity Authorization and Information System—SISBIO.

### Data analysis

It was not possible to know the exact age of nests when they were first found. The earliest record of nests found in this study was October 3rd. Therefore, we fixed 01 October as the starting date of the incubation period for estimating the age of nests used in analyses.

We calculated the probability of predation during the incubation period for nests monitored with camera traps. The maximum incubation period (90 days) was divided into 7-day intervals for analysis. For these analyses, we used only the first predation event for each nest. Temporal clumping of attacks on nests by each kind of predator in the two years of sampling was analyzed using a serial randomness test [20].

To investigate the relationship between female presence and the probability of predation, we only used nests that received at least 3 visits between early October and late December (n = 30). A Fisher's exact test was used to analyze the contingency table.

To evaluate whether some nests were more susceptible to predation than others, we tested whether the proportion of nests with eggs taken by zero, one, two or three species of predator differed from the expected ratios if attacks by each species of predator were independent, using a chi-square test of a contingency table.

To determine if attacks by a species of predator were dependent on previous attacks by another species of predator, we compared the proportions of observed predation with each species acting as the first, second or third predator with a chi-square test of a contingency table.

We compared the mean time between predation events with mean differences when the dates were randomized 999 times to test whether a predation event stimulated subsequent attacks independent of the type of predator.

The total proportions of nests attacked in the two years in which the predators were identified by vestiges were compared to the proportions of nests attacked by different predator species for nests monitored with camera traps, using a Fisher's exact test of the contingency table.

In order to test whether interference by researchers affected the probability of egg predation, we compared the proportion of nests opened for counting and measuring eggs that was attacked by predators with the proportion of unopened nests that were attacked, using a Fisher´s exact test of the contingency table.

## Results

Predation was recorded in 32% of the 658 black caiman nests we monitored in MSDR. The camera traps recorded the species already known to be predators of black caiman eggs (*Panthera onca*, *Tupinambis teguixim*, *Sapajus macrocephalus*), and also the common opossum (*Didelphis marsupialis*) was photographed taking eggs from one nest that had been opened 18 days before for research activity, but not previously attacked by other predators.

**Fig 2:**
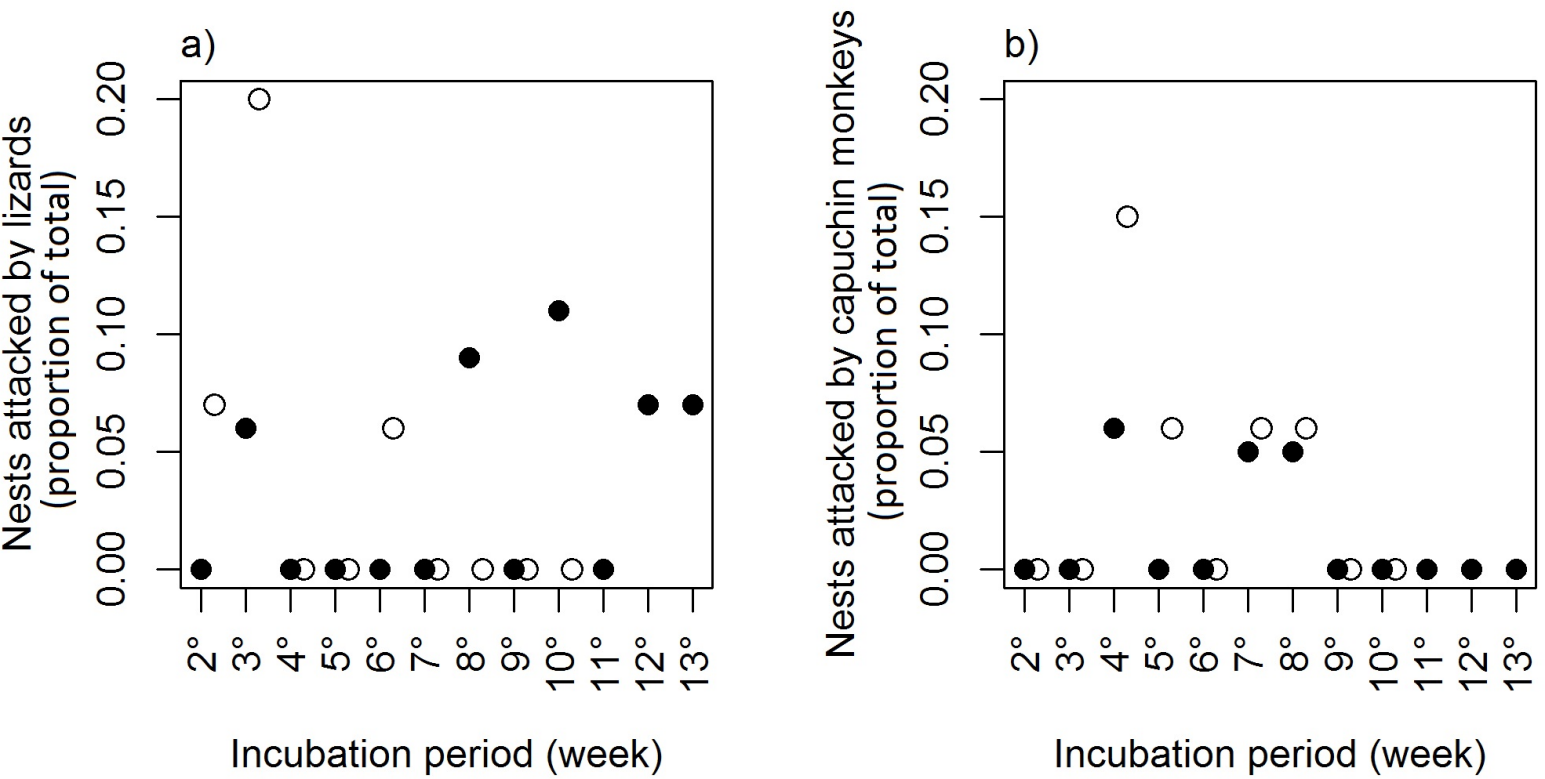
**Relationships between the proportion of black caiman nests attacked by lizards (a) and capuchin monkeys (b) and nest age for nests monitored in 2013 (○) and 2014 (●).** The number of nests available in 2013 in the 2nd to 10th weeks of incubation were 15, 15, 13, 17, 18, 18, 17, 18 and 17, respectively. The number of nests available in 2014 in the 2nd to 13th weeks of incubation were 16, 17, 18, 22, 23, 22, 24, 19, 16, 15, 15, and 15, respectively.

There was no statistically significant relationship (serial randomness test: p> 0.25 in all cases) between the time since the beginning of incubation period and attacks by any of the predator species (Fig 2). Despite the lack of a significant relationship (p = 0.25), predation by capuchin monkeys was concentrated between the fourth and eighth week of incubation (Fig 2B). Attacks on black caiman nests by jaguars were recorded only in one nest in the eighth week of incubation (20 to 26 November) in 2013 and in two nests attacked in the third week (15 to 21 October) in 2014. Data for jaguars were insufficient for statistical tests.

The proportion of nests that were attacked by predators in which we recorded an adult, presumably the female, close to the nest (1 of 30) was significantly lower (Fisher's Exact Test: P = 0.02) than the proportion of nests at which adults were not recorded that were attacked (11 of 30), indicating a lower rate of attack on nests attended by adults.

The probability of a nest being attacked by more than one species of predator was higher than expected by chance if nests were equally likely to be attacked (chi-square test: P = 0.03), indicating that the probability of predation varied between nests.

**Fig 3:**
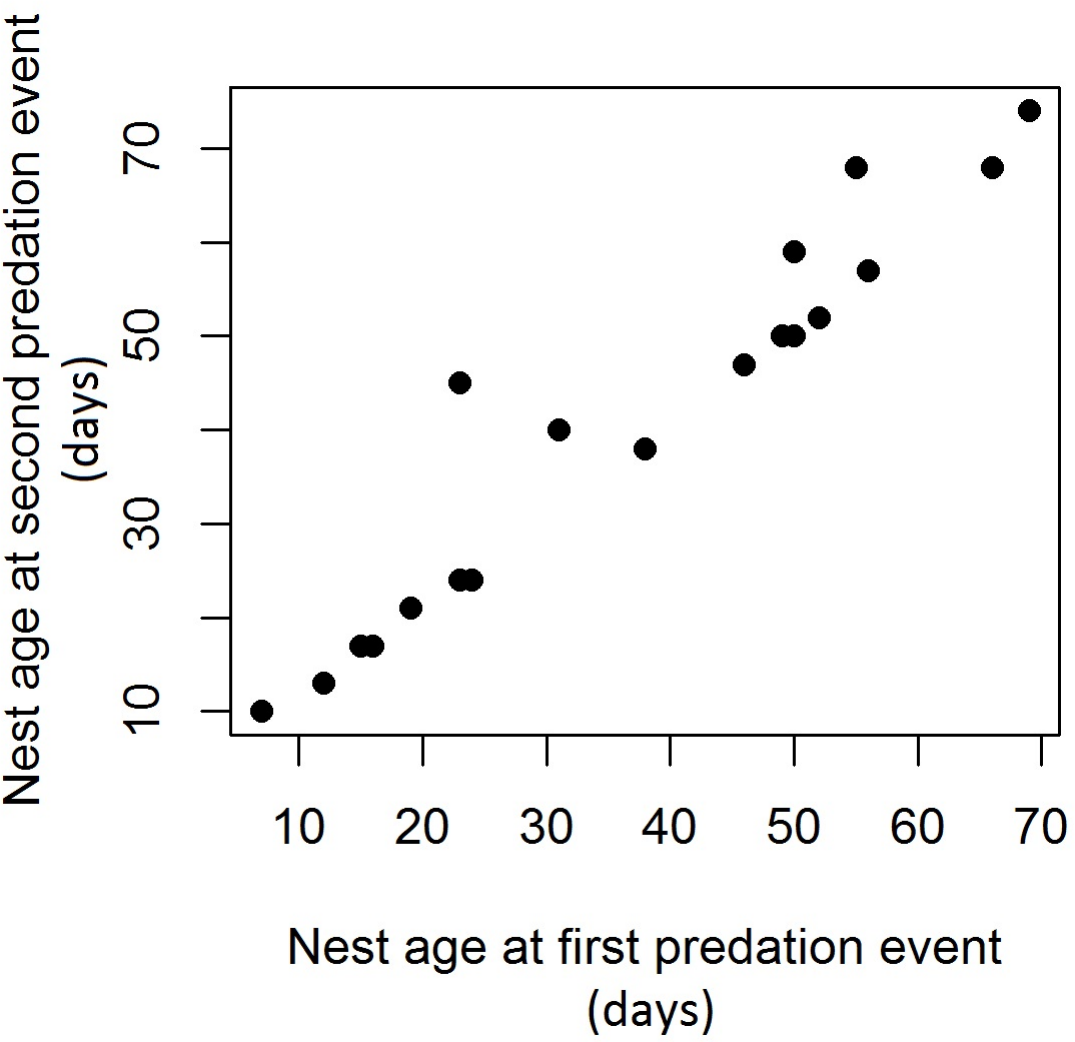
Relationship between the times to first and second predation events in black caiman nests monitored in the years 2013 and 2014.

Occurrence as initial or later predator did not vary between species (chi-square test: P> 0:31), indicating that predation by one species is not necessary for predation by any other species. However, the difference in the age of the nest between the first and second attacks (mean 3.84) was lower than the mean (22.25) expected if the time between the first and second attacks was no greater than expected by chance (P = 0.001), indicating that nest opening in the first predation event facilitated subsequent attacks by the same or other species of predators (Fig 3).

In our study, humans avoided the nests with camera traps, so we cannot compare detection rates for this predator (65 nests monitored by vestiges and only one nest monitored by cameras traps) (Table 1). Humans always took all eggs, so there was no sequential attack for these nests. The proportions of nests attacked by species of predators other than humans estimated from vestiges were different from the proportions of nest predators identified by camera traps when the nest was attacked by only one species of predator (Fisher’s Exact Test: P = 0.04). The proportions of nests attacked by more than one species of predator differed between the two identification methods used (Fisher’s Exact Test: P<0.001). The proportion of nests that were not attacked was similar between methods (0.71 monitored by vestiges and 0.62 monitored by cameras traps) and predators could not be identified for a small proportion (0.02) of nests monitored by vestiges (Table 1). The species identified as nest predators both methods were similar (Fisher’s Exact Test: P>0.05) (Table 2).

**Table 1:** Number and proportion of predators that attacked black caiman nests monitored by vestiges (n = 595) and monitored by cameras (n = 63) in the years 2013 and 2014.

	Vestiges	Cameras
**No predation**	421 (0.71)	39 (0.62)
**1 predator**	74 (0.12)	14 (0.22)
**>1 predator**	22 (0.14)	9 (0.14)
**Unknown**	13 (0.02)	0
**Humans**	65 (0.11)	1 (0.02)

**Table 2:** Number and proportion of nests attack by only one predator other than humans monitored by vestiges (n = 74) and monitored by cameras (n = 14) in the years 2013 and 2014.

	Vestiges	Cameras
**Lizard**	34 (0.46)	5 (0.36)
**Capuchin monkey**	17 (0.23)	6 (0.43)
**Jaguar**	23 (0.31)	2 (0.14)
**Opossum**	0	1 (0.07)

The proportion of nests attacked by predators did not differ statistically between nests that had been opened for research purposes (14 of 63) and nests that had not been opened (49 of 63) for nests monitored by cameras (*Fisher’s Exact Test*: P≈1), indicating that there was little or no effect of research activity on the probability of nest attacks.

## Discussion

The attack rate for predators on black caiman nests recorded in this study (32%) is lower than those recorded in previous studies in the same area. In a study conducted in the Mamirauá Sustainable Development Reserve (MSDR) between 1994 and 1996, eggs in 46% (n = 50) of nests suffered predation [4]. Between 2007 and 2008, 70% (n = 148) of nests in MSDR were attacked by predators [3]. However, the kinds of predators identified were similar in all studies. We also recorded a common opossum attacking a black caiman nest, and that species was not registered in previous studies.

With one exception, we did not register human attacks at nests with cameras, because local people knew that the camera traps were being used to monitor nests. However, vestiges indicated that about 11% of attacks on caiman's nests were by humans.

There was no relationship between nest age and attacks by any of the predator species. Predation on eggs in nests of other species has been related to visual and olfactory attractors that help predators find nests [18,21,22]. We expected more attacks at the beginning of incubation because newly built nests are higher and surrounded by bare ground, which could increase visual detection by predators. It is also likely that females release odors during oviposition, as has been suggested for some species of turtles [21] and water birds [22]. We also expected a higher rate of attacks on nests at the end of incubation because of the possibility that full term embryos were vocalizing in eggs [23,24], which may attract predators. However, our results suggest that the probability of predation on black caiman eggs in the MSDR is relatively constant throughout the incubation period.

Females of many species of crocodilians guard nests during the incubation period, presumably minimizing predator attacks [9,18,25]. Studies in western Ecuador [7] and in MSDR [4,12] reported aggressive behavior of females against humans when defending their nests. Even after a flood that killed all eggs in a nest, a black caiman (presumably the female) attended the nest for a further 15 days [7]. After predation events, female *Alligator mississippiensis* and *Caiman latirostris* reconstruct attacked nests and continue to defend them [26,27].

Our data indicated that predation on eggs in nests in MSDR was lower when adults, presumably females, were present. However, even though camera traps appeared to be effective for recording nest predators, they did not capture all the occasions on which females were close to nests. On some visits, females were seen on nests, but there was no register by the camera trap at that time. Therefore, we could use only data obtained during visits to record the presence of females. The equipment used in this study has range dependent on the temperature of the source in relation to air temperature. The use of photographic equipment with a motion sensor to record the presence of females (eg. [25, 28]) could be used to investigate whether nest defense by females is equally effective against all species of predators. It would be interesting to follow the activities of black caiman females throughout incubation period, as has been done with Amazonian spectacled caimans [11]. Nests of both species are attacked by the same predators. It is feasible that caimans are effective against tegu lizards, capuchin monkeys and opossums, as these are natural prey for species. However, nest defense may be less effective against humans and jaguars, which regularly prey on adult caimans [4].

Nest opening by a predator appeared to increase the chances of a subsequent attack because most repeat attacks on a nest occurred soon after the nest was first opened by a predator. However, an attack by another species does not appear to be necessary to facilitate attacks by other predator species as there was no statistically significant difference between species in the probability of being the first or a subsequent predator. We do not know whether repeated attacks on nests by the same species involved the same individuals, but it is likely that repeated attacks occurred because the predators involved were satiated during the first attack and returned after digesting the previous meal.

Considering only the nests not attacked by humans, the use of vestiges did not result in the same proportion of predators identified for nests attacked for only one predator. When attacked by more than one predator, the vestiges identified only of the last attack. The use of vestiges to identify predators was adequate for identifying the principal predators on eggs in black caiman nests in this study (except for opossum, which attacked only one nest). In many circumstances, the vestiges may be better than the use of camera traps for estimating predation by humans. Despite giving different results than camera traps, this is a low-cost method that could be replicated by local communities in caiman-management areas [29].

All nests of *Caiman crocodilus yacare* in the Pantanal that were subjected to perturbations by researchers were attacked by predators, but only half of the undisturbed nests were attacked [17]. Increased predation on eggs after human interference has also been shown in experiments with *C*. *latirostris* nests in Argentina [18]. An increase of up to 40% was found in predation of eggs in nests of *C*. *crocodilus* that were subject to research activities, such as opening and handling eggs and capture of attending females [9]. In this study, opening nests and handling eggs did not increase the number of attacks on black caiman nests. It is possible that either differences in the degree of care when opening nests, or differences among environments and species were responsible for the lower effect of researcher disturbance in this study.
